# Placental glucocorticoid receptors are not affected by maternal depression or SSRI treatment

**DOI:** 10.1080/03009734.2019.1702126

**Published:** 2020-01-21

**Authors:** Åsa Edvinsson, Angela Hoyer, Malin Hansson, Theodora Kunovac Kallak, Inger Sundström-Poromaa, Alkistis Skalkidou, Susanne Lager

**Affiliations:** a Department of Women’s and Children’s Health, Uppsala University, Uppsala, Sweden;; b Department of Immunology, Genetics and Pathology, Uppsala University, Uppsala, Sweden

**Keywords:** NR3C1, pregnancy, prenatal depression, SSRI, Western blot

## Abstract

**Background:** Prenatal depression is common, with an estimate that up to one in five pregnant women suffers from depressive symptoms. Maternal depression is associated with poor pregnancy outcomes such as preterm birth and low birth-weight. Such outcomes possibly affect offspring development. Previous studies suggest placental RNA levels of the glucocorticoid receptor are altered by maternal depression or anxiety; this stress may affect the placenta of male and female foetuses differently. However, it is unknown if the protein levels and activity of this receptor are additionally affected in women with depressive symptoms or being pharmacologically treated for depression.

**Methods:** In this study, we investigated whether the glucocorticoid receptor (NR3C1) in the placenta is affected by maternal depression and/or selective serotonin reuptake inhibitor (SSRIs) treatment. Placentas from 45 women with singleton, term pregnancies were analysed by Western blot to determine glucocorticoid receptor levels, and by DNA-binding capacity to measure glucocorticoid receptor activation.

**Results:** There were no differences in levels of the glucocorticoid receptor or activity between groups (control, depressive symptoms, and SSRI treatment; *n* = 45). Similarly, there was no difference in placental glucocorticoid receptor levels or activity dependent upon foetal sex.

**Conclusion:** Maternal depression and SSRI treatment do not affect the glucocorticoid receptors in the placenta.

## Introduction

During pregnancy, women experience major physical and psychological changes which increase their risk for development of mental illness. It is estimated that approximately 10–20% of pregnant women experience depressive symptoms (1). The proportion of women suffering from major depressive disorder in pregnancy is lower, but it still affects many women, with a prevalence of about 3–5% (2,3). To manage depression, selective serotonin reuptake inhibitors (SSRIs) are commonly prescribed medications during pregnancy. In Europe, up to 4.5% of pregnant women are prescribed SSRIs (4). In the USA, it is twice as common that women use antidepressant drugs during pregnancy (5). Both maternal depression and SSRI treatment are associated with poor pregnancy outcomes (6–9).

It is common that pregnant women experience anxiety, depression, and psychosocial distress. Such distress often stimulates the hypothalamic–pituitary–adrenal (HPA) axis, resulting in release of stress hormones (for instance cortisol) (10). It has been shown that direct transfer of cortisol from the maternal circulation to the foetus across the placenta is limited (11). But cortisol can also exert its effects through the glucocorticoid receptor (NR3C1) (12), which is present in the placenta (13). Maternal stress has been shown to directly affect the placenta. For instance, increased placental RNA levels of the glucocorticoid receptor have been observed in association with maternal perceived stress or depressive symptoms (14–16). Interestingly, the effects of maternal stress may affect the placenta from female and male infants differently (17,18).

Previous studies have focussed on glucocorticoid receptor mRNA levels, which may not reflect the protein amount of receptor present in the tissue. It is currently unknown if protein levels or activation of the placental glucocorticoid receptor are affected as well. Therefore, the aim of this study was to determine if maternal depression or SSRI treatment affects placental protein levels of glucocorticoid receptor.

## Material and methods

### Study population

Samples utilized in this study are from the Biology, Affect, Stress, Imaging, and Cognition (BASIC) cohort conducted at Uppsala University Hospital, Department of Women’s and Children’s Health (19). The study was approved by the Regional Ethical Review Board in Uppsala (Dnr 2009/171 with amendments). All participants gave informed written consent. Briefly, women who were registered for a routine ultrasound scan at Akademiska University Hospital in Uppsala around 17 weeks gestation were asked about participation in the BASIC study. From participating women, data were collected through web-based surveys, including the Edinburgh Postnatal Depression Scale (EPDS) (20), at 17 and 32 weeks gestation. A sub-set of women was invited to a visit in late pregnancy (around 38 weeks gestation) for a structured psychiatric interview (Mini International Neuropsychiatric Interview, MINI). From the BASIC cohort, 45 women with singleton pregnancies were selected for the present study. The women were divided into three groups: control, depressive symptoms, and SSRI treatment. Women in the control group had a maximum EPDS score of 9 during pregnancy and no history of psychiatric illness according to medical records (*n* = 17). The women in the depressive symptoms group had an EPDS score of 12 or greater in gestational weeks 17 and/or 32 (*n* = 14). Six of these women were depressed according to MINI conducted in late pregnancy. The women in the SSRI treatment group used SSRI medication during at least half of the pregnancy (*n* = 14). All the women in the SSRI treatment group had measurable blood concentrations of their prescribed SSRI at delivery (except one woman prescribed sertraline where no blood sample was available for testing). Clinical characteristics of the selected pregnancies are presented in Table 1.

**Table 1.** Clinical characteristics.

	Healthy control	Depressive symptoms	SSRI treatment
Mother			
*n*	17	14	14
Age at delivery (years)	30 ± 3	31 ± 5	31 ± 5
BMI (kg/m²)	24.9 (21.5–32.2)	23.7 (21.1–26.4)	24.9 (22.5–29.3)
Primiparous women	8 (47%)	6 (43%)	4 (29%)
Preeclampsia	0 (0%)	0 (0%)	0 (0%)
Hypertension	0 (0%)	0 (0%)	0 (0%)
Diabetes	0 (0%)	0 (0%)	0 (0%)
Ethnicity (born in Scandinavia)	17 (100%)	14 (100%)	14 (100%)
College/university education	15 (88%)	10 (71%)	10 (77%)
Missing	0	0	1
Mode of delivery			
Vaginal	13 (76%)	11 (79%)	11 (79%)
Elective CS	1 (6%)	2 (14%)	2 (14%)
Intrapartum CS	3 (18%)	1 (7%)	1 (7%)
EPDS, gestational week 17	2 (1–7)	13 (12–16)	9 (5–12)
Missing	0	0	1
EPDS, gestational week 32	3 (1–6)	15 (14–16)	7 (5–9)
Missing	1	0	2
SSRI	0 (0%)	0 (0%)	Fluoxetine, 5 (36%); Sertraline, 5 (36%); Citalopram, 4 (29%)
Newborn			
Gestational length (days)	281 (276–288)	286 (280–290)	277 (272–280)
Birth-weight (kg)	3.76 ± 0.68	3.90 ± 0.42	3.66 ± 0.38
Sex, proportion female	7 (41%)	7 (50%)	6 (35%)
NICU care after delivery	0 (0%)	0 (0%)	1 (7%)

Data are presented as mean ± SD, median (IQR), or number (%).

BMI: body mass index; CS: Caesarean section; EPDS: Edinburgh Postnatal Depression Scale; IQR: interquartile range; NICU: neonatal intensity care unit; SD: standard deviation; SSRI: selective serotonin reuptake inhibitor.

### Tissue collection

Placental tissue collection was carried out as soon as possible after delivery. A full-thickness biopsy was sampled from the central part of the placenta, selecting areas devoid of calcifications and infarcts. The samples were rinsed in sterile phosphate-buffered saline, frozen on dry ice within 30 min after delivery, and then stored at −70 °C until further processing. From the frozen tissue biopsies, small samples were carefully cut from the villous tissue to exclude tissue from the basal and chorionic plates.

### Western blot

Placental villous tissue samples were homogenized in RIPA buffer (25 mM Tris-HCl pH 7.6, 150 mM NaCl, 1% NP-40, 1% sodium deoxycholate, 0.1% SDS; cat# 89900, Thermo Fisher Scientific) containing Halt Protease Inhibitor Cocktail (cat# 87785, Thermo Fisher Scientific). Protein concentrations of homogenates were determined using the Bradford assay (B6919, Sigma-Aldrich, St. Louis, MO). Western blot was performed on 4–12% Bis-Tris pre-cast gels (NP0323, Thermo Fisher Scientific, Waltham, MA) and transferred onto PVDF membranes (IPFL00010, Merck Millipore, Burlington, MA). After transfer, membranes were stained for total protein with Amido Black Staining Solution (A8181, Sigma-Aldrich) and/or Ponceau S Staining Solution (P7170, Sigma-Aldrich). Total protein stains have been previously proposed as a good reference for Western blotting of placental proteins (21). Thereafter, membranes were blocked for 1 h at room temperature in Odyssey Blocking buffer (927–40000, LI-COR Biosciences, Lincoln, NE). After blocking, the membranes were probed with beta-actin (final concentration 0.2 µg/mL; SC-47778, Santa Cruz Biotechnology, Dallas, TX; 1 h at room temperature) or glucocorticoid receptor (NR3C1; final concentration 81 ng/mL; ab109022, Abcam, Cambridge, UK; overnight at +4 °C). Immunolabeling was visualized with fluorescently labelled secondary antibodies (926–68070 or 926–32211, LI-COR Biosciences) in an Odyssey Sa scanner (LI-COR Biosciences). Images were analysed with ImageJ (version 1.52a). Placental levels of the glucocorticoid receptor (GR) were adjusted for total protein staining intensity (Ponceau S staining).

### Transcription factor activity

Nuclei were isolated from 40 mg of placental villous tissue using the Nuclear Extraction Kit (ab113474; Abcam). Protein concentrations of isolated nuclei were measured with the Bradford assay (Sigma-Aldrich). The DNA binding capacity of NR3C1 was determined with the Glucocorticoid Receptor Transcription Factor Assay Kit (ab207207; Abcam) using 3.9 µg of nuclear extract per well. Nuclei extraction and GR transcription factor assay were performed according to the manufacturer’s instructions. The GR transcription factor activity was tested in duplicate for each placenta.

### Statistics

Statistical analysis was carried out using IBM SPSS Statistics, version 25. Placental glucocorticoid receptor levels and activity were tested for normality using the Shapiro–Wilk test; differences between the groups were evaluated by one-way ANOVA test, *t* test, Kruskal–Wallis, or Mann–Whitney *U* test as appropriate.

## Results

### Western blotting loading control

**Figure 1.**
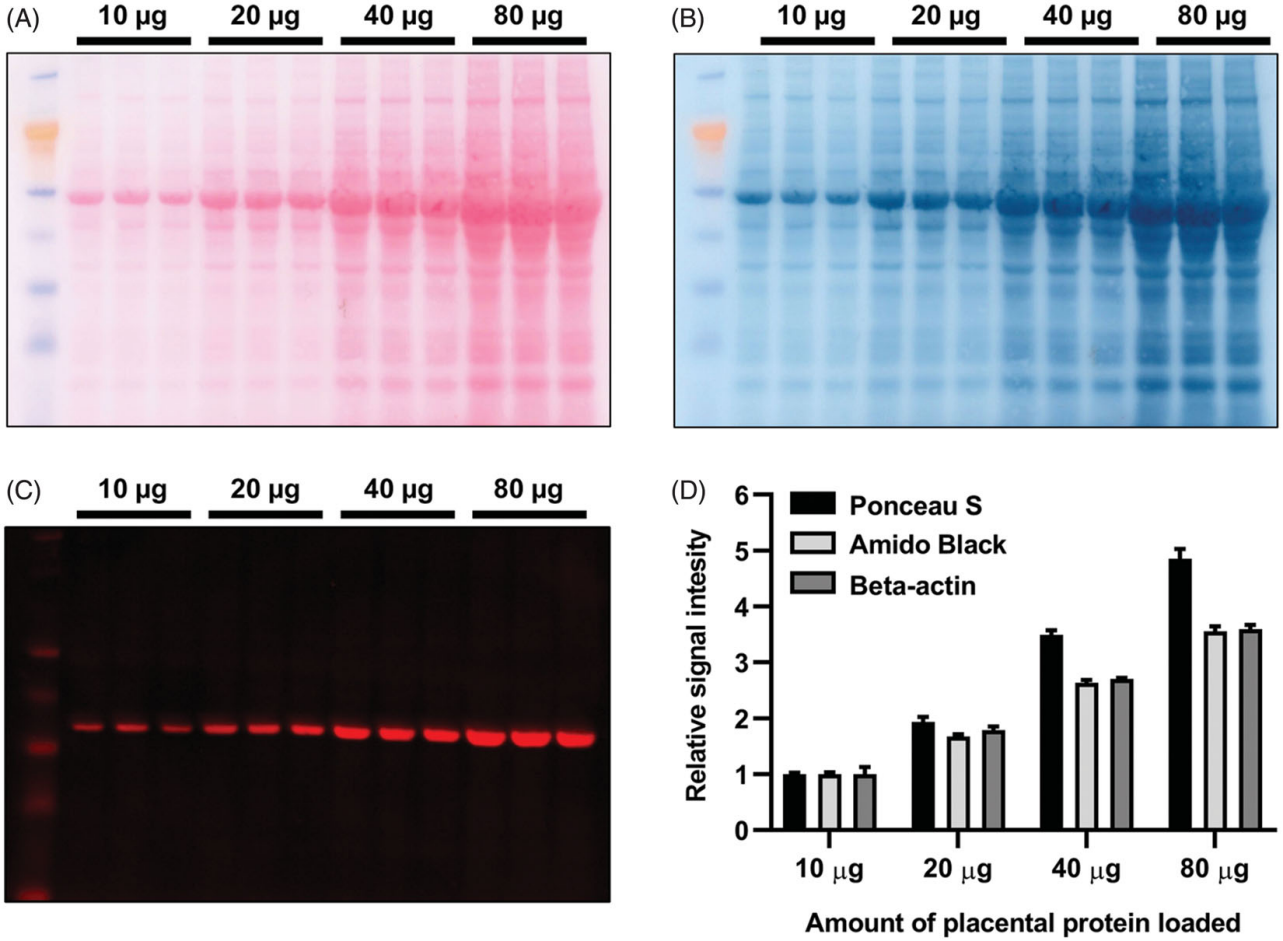
Western blot loading control. For evaluation of loading controls, one membrane was first stained with: (A) Ponceau S; followed by (B) Amido Black; and lastly (C) the membrane was probed for beta-actin. (D) Quantification of total protein stains and beta-actin. Far left lane contained a molecular weight marker. Ponceau S displayed values closest to the expected doubling and was used as loading control for subsequent experiments. The mean values of the 10 µg protein loading signals were assigned a value of 1. Data are presented as mean ± SEM.

Prior to examining placental levels of the glucocorticoid receptor, three different approaches were evaluated as potential loading controls for the Western blot analysis: total protein staining by Amido Black and Ponceau S, as well as probing the membrane for beta-actin (Figure 1). Ponceau S was selected as the appropriate loading control and used in subsequent experiments.

### Placental NR3C1 protein

**Figure 2.**
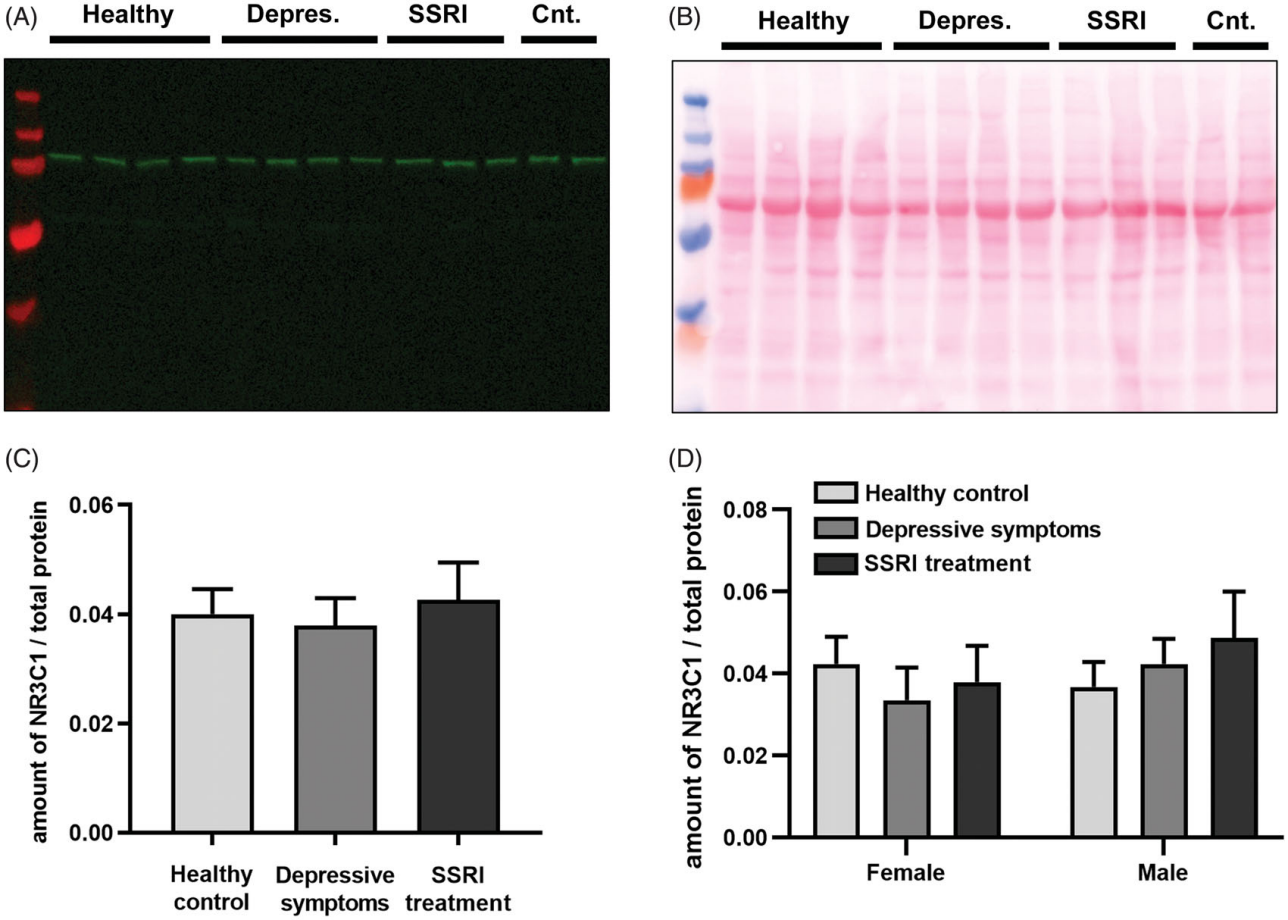
Glucocorticoid receptor protein in the placenta. (A, B) Representative Western blot of NR3C1 in placenta and corresponding Ponceau S stain; 20 µg of placental samples from healthy controls (Healthy), women with depressive symptoms (Depres.), and women with SSRI treatment (SSRI) were loaded on each Western blot gel together with two control samples (Cnt.). The two control samples were loaded on all gels. In the far left lane, a molecular weight marker was loaded. NR3C1 was detected as a main band at ∼100 kDa; a weaker band was detected between 55 and 75 kDa. (C) Quantification of placental NR3C1 (∼100 kDa band) separated into groups (Healthy control, Depressive symptoms, and SSRI treatment). Amount of NR3C1 was adjusted for total protein. *n* = 14–17/group; *p* > 0.05, one-way ANOVA. (D) Foetal sex and placental NR3C1 protein levels. *n* = 6–10/group; *p* > 0.05, one-way ANOVA. Data in graphs are presented as mean ± SEM.

The glucocorticoid receptor was detected as a main band at approximately 100 kDa (Figure 2(A)). In 45 placentas, levels of the glucocorticoid receptor were measured and adjusted for total protein (Ponceau S staining; Figure 2(B)). Detectable level of the glucocorticoid receptor was present in all but two samples. The results were similar when including these two samples (the value of glucocorticoid receptor level as zero) or excluding the samples completely from the analysis. When comparing placentas from healthy controls, women with depressive symptoms, and women using SSRIs during pregnancy, no difference in glucocorticoid receptor levels between the groups was observed (Figure 2(C)). Response to these stressors did not differ depending on foetal sex (Figure 2(D)). Similarly, there was no difference in glucocorticoid receptor levels when comparing placentas from female and male foetuses (mean NR3C1/total protein levels 0.042 ± 0.004 versus 0.038 ± 0.004; *n* = 20–25/group, *p* > 0.05, *t* test).

### Placental glucocorticoid receptor DNA binding activity

**Figure 3.**
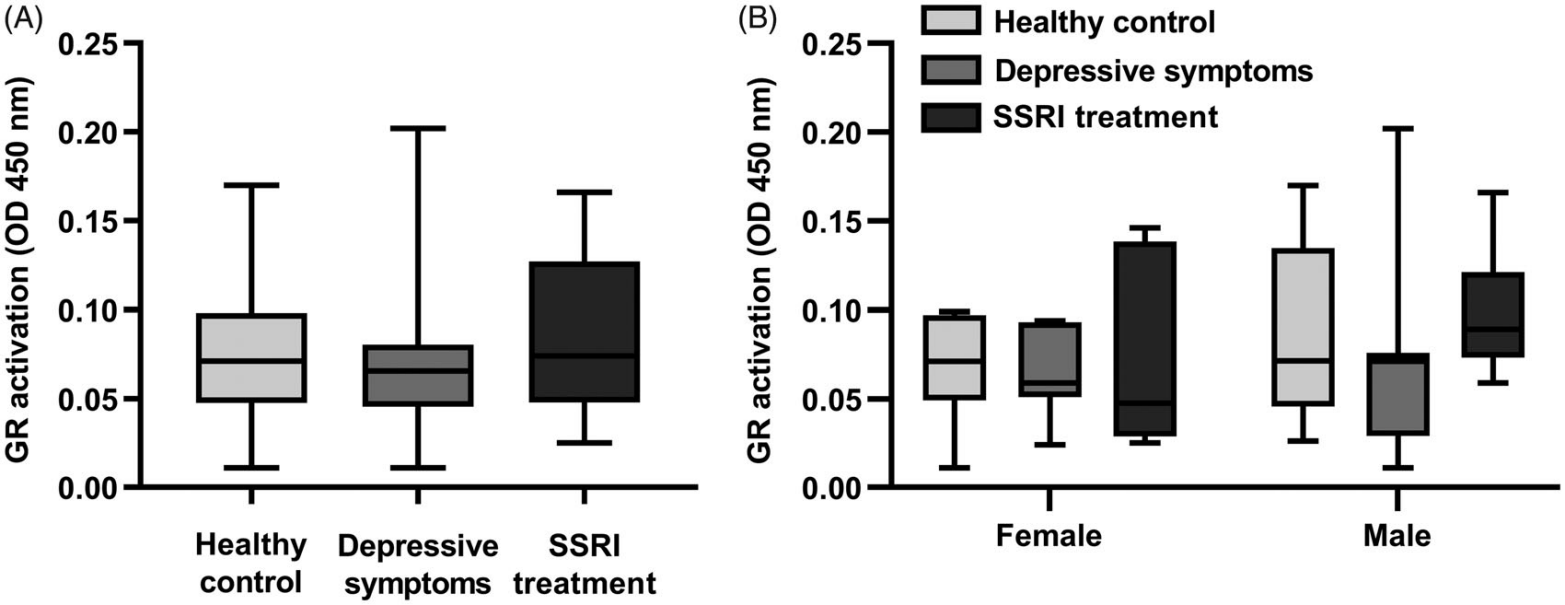
Placental glucocorticoid receptor activity. Activity of the glucocorticoid receptor measured in placental nuclear isolates by DNA binding capacity. (A) Glucocorticoid receptor activity in the three groups of women (Healthy control, Depressive symptoms, and SSRI treatment). *n* = 14–17/group; *p* > 0.05, Kruskal–Wallis. (B) Foetal sex and placental glucocorticoid receptor activation. *n* = 6–10/group, *p* > 0.05, Kruskal–Wallis. Data in graphs are presented as median, interquartile range, and 95% confidence interval.

DNA binding activity of the glucocorticoid receptor was detected in all 45 placentas investigated. There was no difference in glucocorticoid receptor activity between the three groups of women (healthy controls, depressive symptoms, and SSRI treatment; Figure 3(A)). Further, there was no difference in activation of the glucocorticoid receptor relating to foetal sex, neither when comparing placentas from healthy controls, depressive symptoms, and SSRI treatment (Figure 3(B)), nor when comparing placentas from female versus male foetuses (median GR activation 0.057 [95% CI 0.050–0.083] versus 0.074 [95% CI 0.067–0.107]; *n* = 20–25/group, *p* > 0.05, Mann–Whitney *U* test).

## Discussion

In this study, we show that maternal depressive symptoms or use of SSRIs during pregnancy do not affect placental glucocorticoid receptor protein levels. Our finding contrasts with some previous reports describing an association between maternal prenatal stress and higher placental glucocorticoid receptor mRNA levels.

Maternal depressive symptoms or SSRI treatment affects placental RNA levels of multiple genes (22,23). SSRIs can also influence the function of placental cells *in vitro* (24–26). These studies clearly demonstrate that such circumstances (maternal depression and SSRI treatment) affect the placenta. What factors are causative of such alterations need to be determined. However, several differences in circulating factors have been reported with maternal depression or SSRI treatment (27–30). It is possible that one or more of these factors could alter placental RNA and protein levels, including the glucocorticoid receptor.

The human placenta expresses several isoforms of the glucocorticoid receptor (13). For this study, we used an antibody which detects both NR3C1α and NR3C1β isoforms of the glucocorticoid receptor. In our Western blot analysis, one main band for the glucocorticoid receptor isoform was observed. This band likely consists of full-length glucocorticoid receptor α and β. Detecting both isoforms together might be considered a limitation in the current study. However, it is in accordance with many previous studies focussing on placental RNA levels of *NR3C1* (14–16,23,31–34). Furthermore, a strong positive correlation between total *NR3C1* and *NR3C1α* mRNA levels has been reported, a relationship also observed to a lesser extent for *NR3C1β* levels (17). This suggests that total glucocorticoid receptor levels are informative.

We did not observe an effect of maternal SSRI treatment upon placental glucocorticoid receptor levels. This finding is in accordance with a previous study investigating RNA levels by microarray (23). Olivier and co-workers did, however, show that maternal SSRI treatment can affect the placenta, as genes relating to for instance cellular function and maintenance were differentially expressed (23). This suggests that, although SSRIs may have an impact on the placenta, its capacity to respond to glucocorticoids remains.

In this study, we found no effect of maternal depressive symptoms upon placental glucocorticoid receptor levels. Our finding is in accordance with a study by St-Pierre and co-workers, which did not observe an association between maternal depressive symptoms and placental *NR3C1* mRNA levels (17). However, several other studies have suggested that maternal depressive symptoms (15,16) or high perceived stress (14) affect placental glucocorticoid receptor mRNA levels. There may be several possible causes for these divergent results. First, the timing of experienced stress may influence effects on the glucocorticoid receptor. That is, if stress occurs in early, middle, or late pregnancy. Capron (15) estimated depressive symptoms the day before delivery, whereas in our study depressive symptoms were measured at 17 and 32 weeks gestation. Second, degree of stress may also affect the placental glucocorticoid receptor. A continuous low-grade stress may have different effects than a sudden, very stressful event. Finally, the type of stress experienced may also have an impact. Maternal stress can be estimated with a range of different tools, such as EPDS [current study and (15,17)], Centre for Epidemiologic Studies–Depression Scale (16), perceived high stress (14), and experience of a natural disaster (17). Therefore, maternal stress can be viewed as an umbrella term capturing a range of states that may or may not affect cellular signalling in the placenta.

Multiple types of stress experienced by the pregnant mother and the effects upon placental glucocorticoid receptor have been investigated. Some stresses appear to affect placenta from female and male foetuses differently. The stress of experiencing a natural disaster during pregnancy reduces glucocorticoid receptor RNA levels in placentas from male foetuses but not female foetuses (17), whereas high perceived emotional distress in the mother increases *NR3C1α* RNA levels in placentas from female but not male foetuses (18). Furthermore, Mina and co-workers reported that the levels of *NR3C1α* mRNA differ between placentas from female and male foetuses (18). We could not confirm such findings in our study. Neither the response to maternal depressive symptoms and SSRI treatment, nor overall glucocorticoid receptor levels differed depending on foetal sex in our cohort.

Another factor suggested as influencing how the placenta responds to stress is ethnicity. In a study by Capron and co-workers, maternal depressive symptoms were associated with increased placental *NR3C1* RNA levels but only in Caucasian mothers (15). In our study, we did not observe such an association in this cohort of Scandinavian mothers.

It is well established that glucocorticoids are very important for adequate foetal development, but also that excess exposure can have potentially harmful effects, such as growth restriction (35). Glucocorticoids exert numerous effects on placenta, including influence of placental development, regulating placental nutrient transport and release of hormones (36). We report that protein levels of glucocorticoid receptor are unchanged for maternal depressive symptoms or SSRI treatment. Therefore, the placentas retain their capacity to respond to glucocorticoids. This observation is further supported by a similar level of glucocorticoid receptor activation, independent of maternal depressive symptoms or SSRI treatment. An altered sensitivity to glucocorticoids could have major effects on placental function, and subsequently the intrauterine environment, as glucocorticoids can regulate a substantial number of genes in our genome (35).

In conclusion, maternal depression and SSRI treatment do not affect glucocorticoid receptor protein levels or activity in placenta. Further research is needed for confirmation of the effects of prenatal stress upon placental biological mechanisms, as well as additional effects upon offspring.
